# Research on the Allocation of 3D Printing Emergency Supplies in Public Health Emergencies

**DOI:** 10.3389/fpubh.2021.657276

**Published:** 2021-03-26

**Authors:** Jianjia He, Gang Liu, Thi Hoai Thuong Mai, Ting Ting Li

**Affiliations:** ^1^Business School, University of Shanghai for Science of Technology, Shanghai, China; ^2^Super Network Research Center (China), Shanghai, China

**Keywords:** public health emergencies, 3D printing, dispatch of emergency supplies, multi-objective optimization, improved NSGA-II algorithm

## Abstract

Significant public health emergencies greatly impact the global supply chain system of production and cause severe shortages in personal protective and medical emergency supplies. Thus, rapid manufacturing, scattered distribution, high design degrees of freedom, and the advantages of the low threshold of 3D printing can play important roles in the production of emergency supplies. In order to better realize the efficient distribution of 3D printing emergency supplies, this paper studies the relationship between supply and demand of 3D printing equipment and emergency supplies produced by 3D printing technology after public health emergencies. First, we fully consider the heterogeneity of user orders, 3D printing equipment resources, and the characteristics of diverse production objectives in the context of the emergent public health environment. The multi-objective optimization model for the production of 3D printing emergency supplies, which was evaluated by multiple manufacturers and in multiple disaster sites, can maximize time and cost benefits of the 3D printing of emergency supplies. Then, an improved non-dominated sorting genetic algorithm (NSGA-II) to solve the multi-objective optimization model is developed and compared with the traditional NSGA-II algorithm analysis. It contains more than one solution in the Pareto optimal solution set. Finally, the effectiveness of 3D printing is verified by numerical simulation, and it is found that it can solve the matching problem of supply and demand of 3D printing emergency supplies in public health emergencies.

## Introduction

In recent years, there has been a series of public health emergencies around the world, such as SARS, influenza A (H1N1), Escherichia coli outbreaks in Europe, Ebola in West Africa, and COVID-19. In particular, the recent Covid-19 epidemic has basically affected the whole society, including health care, economic structure and social relationships. A global response included countries and states have instituted lockdowns, businesses closed down and some of the supplies appeared to be in a state of shortage. For instance, Personal protective equipment lacks supply capacity. As a result, this leaves both the patient and the caregiver in high-risk situations. Recently, the health and wellness sector has begun to look into how 3DP can help address global needs (1–3).

The outbreak of public health emergencies not only seriously endangers people's health and safety but also has a significant impact on social and economic development. Faced with the outbreak of all kinds of unconventional public health emergencies, it is necessary to realize efficient production plans for emergency supplies. Three-dimensional printing by the method of computer-aided design stratification can use digital information in the production practice, mainly by the method of one-by-one printing to accelerate production, also known as the increase in material manufacturing (4–6). This method has the characteristics of distributed manufacturing and digital manufacturing (7); is applicable to small batches, complex shapes, and customized orders tasks (8); and is widely used in industrial manufacturing, aviation, food, sports, and other fields (9, 10). 3D printing technology is the epitome of “digital intelligent nature,” which has the advantage of one-time molding. The printed products do not need to be assembled again, thus shortening the supply chain, saving a lot of labor, and realizing unmanned production. In the context of public health emergencies, 3D printing is useful, as it can quickly produce medical goggles and respiratory masks. At the same time, in such unique periods, 3D printing technology can also realize unmanned manufacturing, so as to curb the spread of the epidemic. Among the aerospace engineering alliance companies, GKN and Renishaw will provide expertise in 3D printing, according to 3D Science Valley's market watch. Volkswagen has also set up a task force to study how 3D printing can make parts for hospital ventilators and other medical supplies. In addition, a European consortium that includes the engineering firm Leitat, 3D printing firm HP, and medical institutions has tested the prototype LeitAT-1, the first large 3D printing ventilator, which is industrially scalable and could reach production capacity of 50 to 100 units per day within a week. HP, an industrial-scale multi-jet molten 3D printing company in the Leitat ventilator development program, has mobilized the company's in-house 3D printing team and HP digital manufacturing partners to network design, validate, and pro-duce essential parts for medical responders and hospitals, including ventilation valves, breathing filters, and mask fasteners. During the same period, an Irish organization launched the Open Source Ventilator (OSV) project, which aims to develop medical supplies related to the treatment of the COVID-19 outbreak.

Therefore, 3D printing technology can be used to rapidly produce emergency supplies such as masks and goggles, so as to ensure the supply of emergency supplies after disasters. At present, the means of provision of emergency supplies after a disaster include reserve, emergency production, social donation, emergency procurement, international assistance, etc. Among them, the mobilization of stock, social donations, emergency procurement, and international assistance can be seen as the main mobilization of stock. Therefore, many researchers focus on the distribution of emergency supplies (11–15). For example, Ekici et al. (16) established a combination optimization model of epidemic spread and food distribution location selection for the scheduling of various emergency resources, and designed a heuristic algorithm to solve large-scale practical problems. He and Liu (17) designed the emergency demand prediction model and the unmet demand penalty function based on the SEIR model, and established a linear programming model of the epidemic emergency logistics network based on this. Liu and Zhang (18) constructed an inter-active coordination optimization model for dynamic ordering and distribution of medical resources among hospitals, distribution centers, and suppliers in the context of influenza spread. Zhan et al. (12) developed a multi-objective optimization model based on disaster scenario information update with the aim of tackling the emergency distribution problem of multiple suppliers, multiple disaster sites, multiple emergency materials, and multiple vehicles in emergency logistics. Alem et al. (15) constructed new two-stage random network traffic models and incorporated the characteristics of procurement, vehicles, lead time, and budget allocation into the models. Scholars have achieved fruitful results in the study of emergency supply distribution. However, if the mobilization of all stocks fails to meet the needs of the affected areas, policy makers need to consider emergency production as a means of securing emergency supplies. For example, after an earthquake in China, in order to meet the living needs of the people in the affected areas, aided provinces were required to carry out production tasks. Although the emergency production tasks of mobile plank houses were successfully completed in the end, many problems also occurred during the implementation process. For example, some places did not have the production capacity or prefabricated houses, while production carried out far away from the quake-hit points suffered from limited coordination and optimization of resource use due to the necessity of adopting different modes of production and sending personnel to different areas. Therefore, many researchers also focus on the utilization of capacity reserve to solve the problem of emergency production. Whybark (19) pointed out that the balance between inventory reserve and emergency production capacity reserve should be considered, and production capacity reserve can effectively reduce the inventory level. In addition to the above research on the production capacity reserve of emergency supplies, Chakravarty (20) focused on the rapid response of humanitarian relief supply chains under uncertain conditions and determined the supply and supply time of emergency supplies after disasters. Sheu and Pan (21) established a stochastic dynamic programming model of material supply through a two-layer recursive function. Wang et al. (22) used option contracts to coordinate the emergency supply chain and realized Pareto improvement in order to solve the problem of shortage of emergency supplies and high purchase prices. Therefore, how to match 3D printing emergency supplies with 3D printing equipment for production and maximize the cost and time benefit of disaster-affected users has become an urgent problem to be solved. Luo et al. (23) is based on the cloud manufacturing paradigm, this study focuses on dynamic and static data based matching method for cloud 3D printing.

Many scholars have studied the scheduling of 3D printing tasks, and there are many effective scheduling methods. However, at present, some scholars only consider one goal, while users generally consider multiple factors comprehensively when choosing 3D printing services. In addition, a few scholars considered a variety of factors, but these scholars generally used the method of additional weight to transform multiple targets into a single target for solving, and the method of additional weight is subjective to a certain extent. Therefore, under full consideration of emergent public health events in relation to the 3D printing of emergency supplies and their characteristics, as well as the cost and time needed for their production, a combined scheduling model of emergency supplies is established, and it is solved with the improved NSGA-II algorithm and multiple non-inferior solution set. After the contribution of this paper is to improve the NSGA-II algorithm is applied to by 3D printing equipment in the production of emergency supplies distribution field. At the same time, the multi-objective optimization problem is also solved.

The remainder of this article is organized as follows: the second part discusses the 3D-printed emergency material scheduling model in the context of public health emergencies; the third part provides an improved NSGA-II algorithm; in the fourth part, an example is given to verify the effectiveness of the proposed method; finally, the fifth part provides the conclusion and briefly discusses further research work.

## Scheduling Model of 3D-Printed Emergency Supplies in the Context of Public Health Emergencies

### Problem Description and Model Assumptions

After an emergency occurs, emergency reserve is the preferred way to raise emergency supplies. When supplies are in short supply, they can also be supplemented by direct requisition, market procurement, organization donation, and other fund-raising methods. However, after a major emergency (especially a major natural disaster) occurs, due to the large area and large population affected by the disaster, the emergency supplies collected only by the above means may not be able to meet the emergency needs; thus, relevant manufacturers need to be urgently mobilized for emergency production. In order to successfully complete the emergency production task, manufacturers with a strong emergency material production capacity and close to the disaster site should be selected. If the raw material reserves of manufacturers are insufficient, suppliers with a strong raw material supply capacity and close to the manufacturers should also be selected as the manufacturers. The purpose of this paper is to build a mathematical model considering the above conditions and provide an emergency production plan and emergency material supply plan with the shortest task times and the lowest costs. Due to the complexity of the production situation in emergency situations, the following assumption are proposed:

(1) The time and unit transportation costs of materials delivered by each 3D printing supplier to each affected user are known;
(2) The production capacity per unit time of 3D printing manufacturers is known;
(3) After all emergency supplies are produced by 3D printing manufacturers, they will transport them to the affected users.

### Mathematical Model

For*I*manufacturing tasks *T*_1_, *T*_2_, *T*_3_,$T_{i}^{,}$ and*J*manufacturing service providers *M*_1_, *M*_2._ *M*_3_, *M*_*j*_, the multi-objective function and constraint mathematical model are used to describe the 3D printing emergency material scheduling in public health emergencies. In this article, we assume that a print-type manufacturing service can only perform subtasks of a specific print type, and that a manufacturer can receive multiple subtasks of the same type simultaneously. Table 1 shows the definitions of the symbols used in the integrated scheduling model.

**Table 1:** Main mathematical notations.

**Notation**	**Definition**
*J*	Total number of 3D printing services
*M*_*j*_	The i-th 3D printing service
*X*_*j*_	The abscissa of the position of *M*_*j*_
*Y*_*j*_	The ordinate of the position of *M*_*j*_
*U*_*j*_	The length of *M*_*j*_
*V*_*j*_	The width of *M*_*j*_
*W*_*j*_	The height of *M*_*j*_
*S*_*j*_	The printing material type of *M*_*j*_
*P*_*j*_	The printing accuracy of *M*_*j*_
${M_{j}}^{c}$	Printing cost of unit task on *M*_*j*_
${M_{j}}^{k}$	Printing speed of unit task on *M*_*j*_
*I*	Total number of 3D printing Order
*T*_*i*_	The i-th 3D printing Order
*x*_*i*_	The abscissa of the position of*T*_*i*_
*y*_*i*_	The ordinate of the position of *T*_*i*_
*u*_*i*_	The length of *T*_*i*_
*v*_*i*_	The width of *T*_*i*_
*w*_*i*_	The height of *T*_*i*_
*s*_*i*_	Required material type of *T*_*i*_
*p*_*i*_	Required accuracy of *T*_*i*_
${T_{i}}^{w}$	The Weight of *T*_*i*_
α	Logistic time between unit distance
β	Logistic cost of unit task between unit distance

### Objective Function

(1) We assume that there are multiple disaster-affected users with different needs for 3D printing emergency supplies at the same time. The order set of 3D printing emergency supplies is *T* = {*T*_1_, *T*_2_, ⋯, *T*_*i*_}, and the set of 3D printing equipment is *M* = {*M*_1_, *M*_2_, ⋯, *M*_*j*_}. The first goal of the scheduling model for 3D-printed emergency material in public health emergencies is to minimize the cost of emergency materials. In the formula, the cost of emergency supplies is composed of the printing cost of each item of 3D printing equipment and the transportation cost in the logistics process. The transportation cost is the sum of the product of the distance between the affected user and service provider and the logistics cost factor. Its objective function is as follows:

(1) $$minf_{1\min}=\sum_{i=1}^{i}\sum_{j=1}^{j}\left[\begin{array}{l} x_{t}^{j}T_{i}^{w}M_{j}^{c}\\ +u_{i}v_{i}w_{i}\beta \sqrt{{\left(x_{i}-X_{j}\right)}^{2}+{\left(y_{i}-Y_{j}\right)}^{2}} \end{array}\right]$$

(2) The second goal of the scheduling model for 3D-printed emergency supplies in public health emergencies is to minimize the time of emergency supplies. In the formula, the time of emergency supplies is composed of the processing time of each item of 3D printing equipment and the transportation time in the logistics process. The transportation time is the sum of the distance between the affected demander and service provider and the logistics speed factor. Its objective function is:

(2) $$\begin{array}{l} minf_{2\min}=\overset{I}{\underset{i=1}{\sum }}\overset{J}{\underset{j=1}{\sum }}\left[x_{i}^{j}\frac{T_{i}^{w}}{M_{j}^{k}}+\frac{\sqrt{{\left(x_{i}-X_{j}\right)}^{2}+{\left(y_{i}-Y_{j}\right)}^{2}}}{\alpha }\right] \end{array}$$

### Constraints

(3) $$\begin{array}{l} u_{i}\leq U_{j} \end{array}$$

(4) $$\begin{array}{l} v_{i}\leq V_{j} \end{array}$$

(5) $$\begin{array}{l} w_{i}\leq W_{j} \end{array}$$

(6) $$\begin{array}{l} s_{i}=S_{j} \end{array}$$

(7) $$\begin{array}{l} P_{j}\leq p_{i} \end{array}$$

(8) $$\{\begin{array}{l} 1\leq i\leq I\\ 1\leq j\leq J \end{array}$$

Equation (3), (4), and (5) indicate that the length, width, and height of the order task are less than or equal to the length, width and height of the 3D printing device, respectively; Equation (6) indicates that the type of the 3D printing device is the same as the required device type of the order; Equation (7) indicates that the printing accuracy of the 3D printing device is less than or equal to the accuracy required by the order; Equation (8) indicates that the order number and the 3D printing equipment number are less than or equal to the order number and the 3D printing equipment number, respectively.

### Decision Variables

$x_{i}^{j}$ is a variable of 0–1. If the i-th order matches the k-th 3D printing device, then; otherwise, it is 0.

## Solving the Multi-Objective Optimization Model

The NSGA-II algorithm (24) introduces a fast non-dominated sorting method, which reduces the computational complexity and has an elite retention strategy. Using crowding distance instead of specifying shared parameters improves the computational efficiency and is a better algorithm to solve multi-objective optimization problems. In the crossover process of the former non-dominated sorting genetic algorithm (NSGA-II), the simulated binary crossover operator (SBX) is commonly used to generate offspring. However, on the surface of a large number of studies, the SBX method cannot avoid the problems of a limited search scope and instability in the evolution process in some cases. At the same time, the NSGA-II algorithm is prone to premature and other phenomena in the optimization process. When directly applied, it often leads to uneven distribution of Pareto front.

### Improvement of NSGA-II

In this paper, an improved NSGA-II algorithm is used to solve the scheduling model for 3D printing tasks. This method is improved by two aspects based on the traditional non-dominated sorting genetic algorithm.

### Introducing the Normal Distribution Crossover Operator (NDX)

The NSGA-II algorithm operating SBX operators are adopted to simulate the evolution process of binary crossover operator, individual creation way as shown in type (9), *a* is a random variable, because of the search scope is limited, easy to appear the problem such as local optimum and evolutionary process is not stable. In view of the deficiency of SBX operator, the normal distribution is introduced into the crossover operator SBX, That is 1.481 replaces *a* to expand the search space, as shown in Equation (10). In order to further enhance the spatial search capability, the discrete recombination operation of evolutionary strategy is introduced into Equation (11).

In this paper, the NDX operator is introduced into the NSGA-II algorithm. The Pareto optimal solution can be uniformly extended to the whole Pareto domain, ensuring the diversity of the population and improving the quality of the Pareto optimal solution. This algorithm is applied to 3D printing task scheduling, which not only pro-vides as many representative non-inferior solutions as possible for the 3D printing service platform but also facilitates it to make more reasonable decisions.

(9) $$\begin{array}{l} o_{1,i}=\frac{p_{1,i}+p_{2,i}}{2}\pm \frac{a\left(p_{1,i}-p_{2,i}\right)}{2} \end{array}$$

Assuming the parent generation and the NDX is used to generate the child generation *o*_1_and . For the *i* variable, the crossover process is as follows: a random number *r* ∈ (0, 1) is generated

(10) $$\begin{array}{l} When\;r\leq 0.5.....o_{1,i}=\frac{p_{1,i}+p_{2,i}}{2}\pm \frac{1.481\left(p_{1,i}-p_{2,i}\right)|N\left(0,1\right)|}{2} \end{array}$$

(11) $$\begin{array}{l} When\;r\leq 0.5.....o_{1,i}=\frac{p_{1,i}+p_{2,i}}{2}\mp \frac{1.481\left(p_{1,i}-p_{2,i}\right)|N\left(0,1\right)|}{2} \end{array}$$

Type:*o*_1,*i*_ is the *i* th variable on the progeny chromosome; *p*_1,*i*_ and *p*_2,*i*_ is the *i* th variable on the paternal chromosome. When|*N*(0, 1)|is a normal distribution random variable.

### Improvement of Congestion Distance in the NSGA-II Algorithm

**Figure 1:**
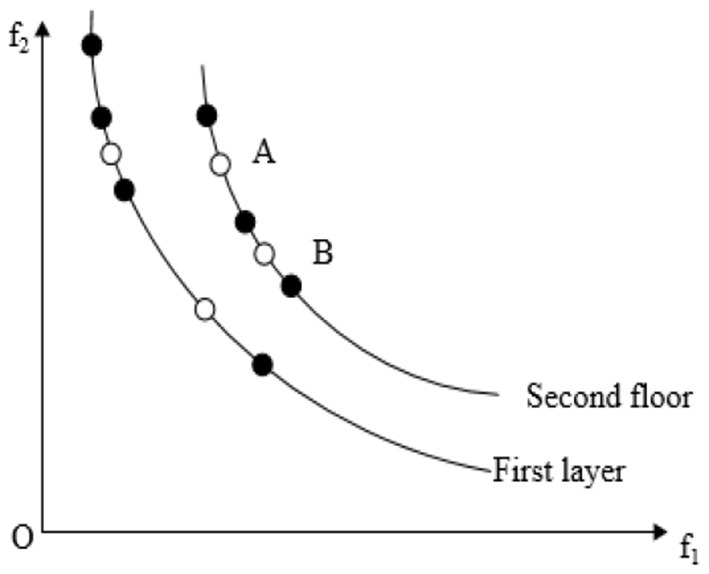
Calculation method of individual crowded distance in the NSGA-II algorithm.

Crowding distance has an important influence on the diversity of the population of the NSGA-II algorithm. Good population diversity not only enables individuals to have good uniform distribution characteristics but also keeps a certain distance between individuals. f_1_and f_2_are the two objective functions of the problem as showed in Figure 1. After the N th iteration using the NSGA-II algorithm, the distribution of individuals in the population is as shown in Figure 1, where individuals *Z*_*i*−1_, and *Z*_*i*+1_are all non-dominated solutions of the first layer. Then, the crowding distance of the individual is the sum of the distances between the former *Z*_*i*−1_and the latter *Z*_*i*+1_ on the two objective functions, which is *a*+*b*. In order to ignore the difference between different objective function value domains, it is also necessary to divide the difference between the extreme points in this hierarchy.

From this, it can be concluded that when there are k objective functions, the crowding distance *d*[*Z*_*i*_] of individual is calculated by Equation (12), and the distribution among individuals is evaluated based on this.

(12) $$\begin{array}{l} d\left[Z_{i}\right]\text{=}\overset{K}{\underset{m=1}{\sum }}\frac{\left|f{\left(Z_{i+1}\right)}_{m}-f{\left(Z_{i-1}\right)}_{m}\right|}{f_{m}^{\max}-f_{m}^{\min}} \end{array}$$

where m is the dimension of the target; *f*(_*Z*_*i*+1_)*m*_ and *f*(_*Z*_*i*−1_)*m*_ are the fitness values of the *Z*_*i*+1_ and *Z*_*i*−1_ individuals to the m-th target, respectively and $f_{\min}^{m}$ are the maximum and minimum fitness values of the M th target in the same distribution layer, respectively.

**Figure 2:**
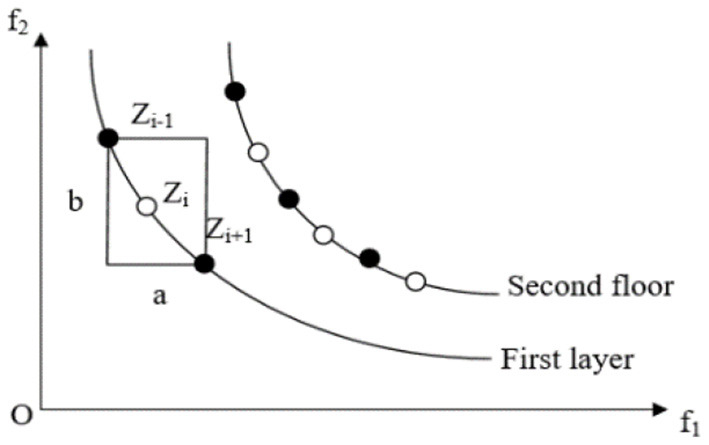
Population individual distribution map.

The introduction of crowding distance can make the distribution of the optimal solution more uniform and maintain the diversity of the population. As shown in Figure 2, however, this strategy also has some drawbacks:

(1) If the number of individuals in the current non-dominated set is greater than the specified population number, the surplus individuals are eliminated at one time according to the crowding distance from small to large. This strategy is somewhat crude, as only one population maintenance process needs to calculate the crowding distance of individuals once. If all individuals with small crowding distance are eliminated at one time, there will be individual deletions among individuals, making the distribution of solutions poor.

(2) At the same level, when point A and point B are relatively crowded, point B is eliminated according to the exclusion mechanism of the current NSGA-II algorithm, thus preserving point A. However, in the actual whole population distribution, because there are more individuals in the upper layer around A, it is not necessarily the best selection strategy to keep point A. It can be seen that the crowding mechanism based on crowding distance cannot view the density around individuals from the perspective of the whole population distribution.

With the aim of shortening the congestion distance mentioned above, this paper proposes a diversity maintenance strategy called dynamic congestion distance. For the first deficiency, this paper adopts the following strategy: in the process of population maintenance, the remaining crowding distance in the population is recalculated for each individual eliminated. For the second deficiency, the dynamic congestion distance of individual*Z*_*i*_ is calculated according to the following formula:

(13) $$\begin{array}{l} dd\left[Z_{i}\right]=\frac{d\left[Z_{i}\right]}{\mathrm{lg}\left(1/R_{i}\right)} \end{array}$$

Where $R_{i}=\frac{1}{m}\overset{K}{\underset{m=1}{\sum }}{\left(|f{\left(Z_{i+1}\right)}_{m}-f{\left(Z_{i-1}\right)}_{m}|-d\left[Z_{i}\right]\right)}^{2}$ is the variance of crowding distance between individuals *Z*_*i*_ adjacent to targets in all dimensions. The larger the variance, the sparser the distribution among individuals but the closer the distribution. Through the dynamic crowding distance method, individuals with small difference values are eliminated, and individuals with large difference values are retained, thus greatly improving the diversity of the algorithm population.

### Improvement to the Execution Process of the NSGA-II Algorithm

The improvement of the NSGA-II algorithm is achieved with the following steps:

Step 1: In the 3D printing emergency resource scheduling, each 3D printing emergency material *T*_*i*_ corresponds to an alternative set of 3D printing equipment*M*_*j*_allowing real number coding to be adopted. The coding rules are as follows: each 3D printing emergency material *T*_*i*_ is numbered 1, 2, 3, etc. t; each chromosome of the genetic algorithm is composed of t genes; and each gene represents a 3D-printed emergency supply. There are m items of 3D printing equipment in the form of service providers, and t 3D printing emergency orders are allocated to m 3D printing equipment. In the process of scheduling emergency supplies, each item of 3D printing equipment can produce multiple 3D printing emergency supplies, but each 3D printing emergency supply can only be allocated to a single piece of 3D printing equipment.

**Figure 3:**
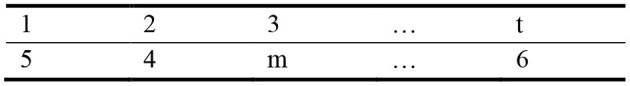
Chromosome coding diagram.

The value on each gene of the initial individual is randomly generated from the alternative 3D printing equipment*M*_*j*_ for 3D printing emergency supplies. The encoded chromosome structure is shown in Figure 3. The gene position represents distributed 3D printing emergency supplies *T*_*i*_ and the gene value represents the 3D printing equipment produced by it as shown in Figure 3.

Step 2: The initial population of N individuals is randomly generated under the constraint conditions of Equations (3)–(7), and the evolutionary algebra *n* = 0.

Step 3: According to fitness functions (1) and (2), fast non-dominant sorting was conducted for all population individuals, and the crowding degree of each population individual was calculated.

Step 4: Randomly selected individuals in population*P*_*n*_ were selected by the binary championship selection operation, crossover of NDX operators, and mutation to produce offspring *X*_*n*_.

Step 5: *P*_*n*_ and *X*_*n*_are merged to produce *Q*_*n*_and calculate the value of the objective function, and a quick non-dominant sort operation on *Q*_*n*_is performed.

Step 6: By calculating the crowding degree and crowding distance of individuals in *Q*_*n*_ (adopting the improved crowding distance mentioned above), the optimal N individuals are formed into a new generation population *P*_*n*+1_.

**Figure 4:**
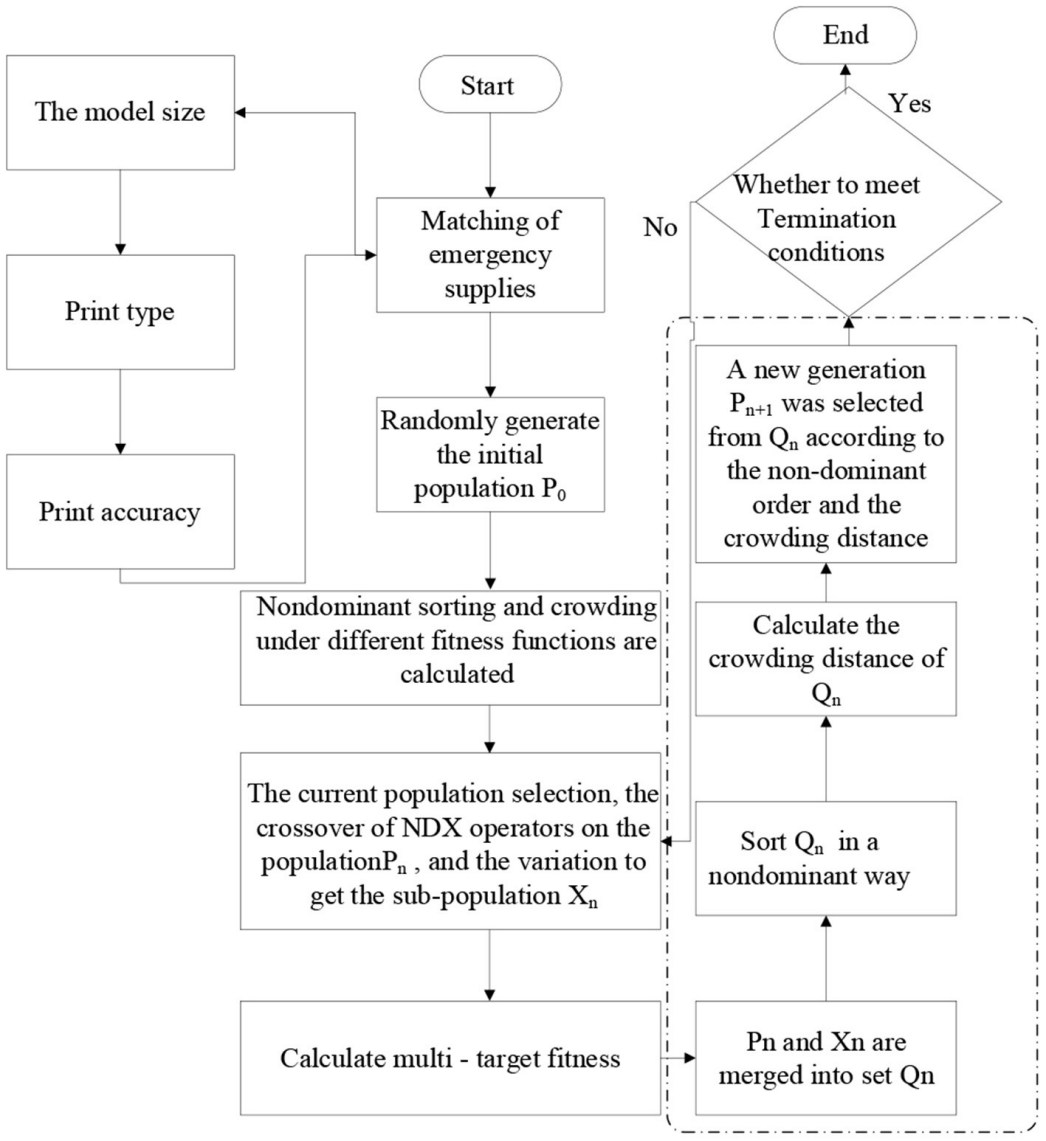
Improved NSGA-II flow chart.

The improved NSGA-II algorithm was used to solve the scheduling flow diagram of the distributed 3D printing emergency supplies, as shown in Figure 4.

## Example Analysis

### Example Description and Parameter Setting

The feasibility and validity of the model and algorithm are verified by an example. The parameter settings are shown in Table 2. The manufacturer has 20 pieces of 3D printing equipment, and the equipment information is shown in Table 3. We assumed that there are 50 different 3D printed emergency supplies from the affected demanders, and the specific information of the emergency supplies is shown in Table 4.

**Table 2:** Experimental parameter setting.

**Parameter**	**Value range**
*J*	20
*I*	50
α	20 km/h
β	3.5¥/km

**Table 3:** 3D printing device information.

**ID of 3D printer**	***S*_*j*_**	***P*_*j*_(*mm*)**	${M_{j}}^{c}($¥**/*g*)**	**${M_{j}}^{k}\left(g/h\right)$**	***U*_*j*_(*m*)**	***V*_*j*_(*m*)**	***W*_*j*_(*m*)**	***X*_*j*_(*km*)**	***Y*_*j*_(*km*)**
1	FDM	0.01	1.5	70	0.69	0.78	0.62	60	200
2	FDM	0.02	1.4	74	0.63	0.68	0.51	180	200
3	FDM	0.05	1.1	62	0.85	0.87	0.75	80	180
4	3DP	0.06	0.7	78	0.52	0.63	0.54	140	180
5	SLA	0.01	1.7	68	0.66	0.75	0.98	20	160
6	SLA	0.02	1.6	70	0.72	0.95	0.63	100	160
7	SLS	0.05	1.2	70	0.46	0.39	0.63	200	160
8	SLS	0.03	1.3	68	0.75	0.65	0.56	140	140
9	FDM	0.03	1.3	62	0.86	0.74	0.46	40	120
10	FDM	0.04	1.2	63	0.49	0.46	0.67	100	120
11	SLA	0.02	1.2	74	0.83	0.94	0.86	180	100
12	3DP	0.02	0.9	62	0.91	0.94	0.74	60	80
13	3DP	0.05	0.6	78	0.71	0.68	0.91	120	80
14	SLS	0.02	1.4	69	0.45	0.36	0.79	180	60
15	SLS	0.03	1.5	71	0.65	0.58	0.52	20	40
16	FDM	0.04	1.2	63	0.49	0.46	0.67	120	120
17	3DP	0.02	0.9	62	0.91	0.94	0.74	160	180
18	SLA	0.02	1.2	74	0.83	0.94	0.86	140	100
19	SLS	0.02	1.4	69	0.45	0.36	0.79	180	60
20	SLS	0.03	1.5	71	0.65	0.58	0.52	20	40

**Table 4:** 3D printing order information.

**ID of the task**	***S*_*i*_**	***P*_*i*_(*mm*)**	${T_{i}}^{w}\left(g\right)$	***u*_*i*_(*m*)**	***v*_*i*_(*m*)**	***w*_*i*_(*m*)**	***x*_*i*_(*km*)**	***y*_*i*_(*km*)**
1	FDM	0.03	1,600	0.31	0.33	0.63	100	110
2	SLA	0.02	1,800	0.38	0.42	0.32	100	110
3	SLS	0.06	1,800	0.62	0.63	0.74	100	110
4	FDM	0.05	1,100	0.53	0.53	0.46	100	110
5	SLS	0.05	800	0.36	0.37	0.52	100	110
6	SLA	0.01	1,300	0.36	0.37	0.25	100	120
7	SLS	0.06	1,200	0.39	0.40	0.48	100	120
8	SLA	0.04	1,600	0.17	0.39	0.30	100	120
9	3DP	0.06	1,200	0.48	0.39	0.30	100	120
10	FDM	0.04	1,300	0.28	0.29	0.30	100	120
11	3DP	0.02	700	0.58	0.59	0.73	120	110
12	3DP	0.03	900	0.38	0.29	0.25	120	110
13	3DP	0.03	300	0.48	0.39	0.35	120	110
14	FDM	0.06	600	0.67	0.69	0.30	120	110
15	SLA	0.02	1,700	0.32	0.34	0.68	120	110
16	FDM	0.06	1,800	0.25	0.23	0.55	120	120
17	FDM	0.02	1,800	0.58	0.52	0.51	120	120
18	FDM	0.03	1,400	0.28	0.31	0.30	120	120
19	SLA	0.02	1,300	0.66	0.39	0.50	120	120
20	3DP	0.05	1,200	0.28	0.26	0.28	120	120
21	FDM	0.03	800	0.35	0.29	0.42	130	100
22	SLA	0.06	900	0.51	0.38	0.5	130	100
23	SLA	0.04	1,100	0.29	0.35	0.62	130	100
24	FDM	0.03	1,000	0.16	0.26	0.27	130	100
25	SLS	0.04	1,100	0.18	0.51	0.38	130	100
26	3DP	0.06	1,200	0.36	0.27	0.39	130	110
27	3DP	0.02	1,800	0.37	0.29	0.74	130	110
28	SLS	0.03	1,600	0.39	0.41	0.38	130	110
29	FDM	0.02	600	0.35	0.38	0.38	130	110
30	SLS	0.06	1,500	0.42	0.37	0.37	130	110
31	SLA	0.03	1,700	0.47	0.19	0.67	140	120
32	SLS	0.05	800	0.35	0.24	0.56	140	120
33	3DP	0.06	600	0.16	0.28	0.34	140	120
34	FDM	0.03	500	0.63	0.36	0.64	140	120
35	SLA	0.03	200	0.61	0.34	0.37	140	120
36	3DP	0.04	800	0.62	0.37	0.46	150	110
37	FDM	0.03	700	0.35	0.28	0.47	150	110
38	SLA	0.04	700	0.35	0.28	0.47	150	110
39	FDM	0.03	1,600	0.38	0.41	0.52	150	110
40	FDM	0.04	1,400	0.37	0.43	0.36	150	110
41	FDM	0.05	1,100	0.53	0.53	0.46	100	110
42	FDM	0.05	1,100	0.53	0.53	0.46	100	110
43	SLS	0.05	800	0.36	0.37	0.52	100	110
44	SLA	0.01	1,300	0.36	0.37	0.25	100	120
45	SLS	0.06	1,200	0.39	0.4	0.48	100	120
46	SLA	0.04	1,600	0.17	0.39	0.3	100	120
47	3DP	0.06	1,200	0.48	0.39	0.3	100	120
48	FDM	0.04	1,300	0.28	0.29	0.3	100	120
49	3DP	0.02	700	0.58	0.59	0.73	120	110
50	3DP	0.03	900	0.38	0.29	0.25	120	110

### Example Results and Solution Analysis

**Figure 5:**
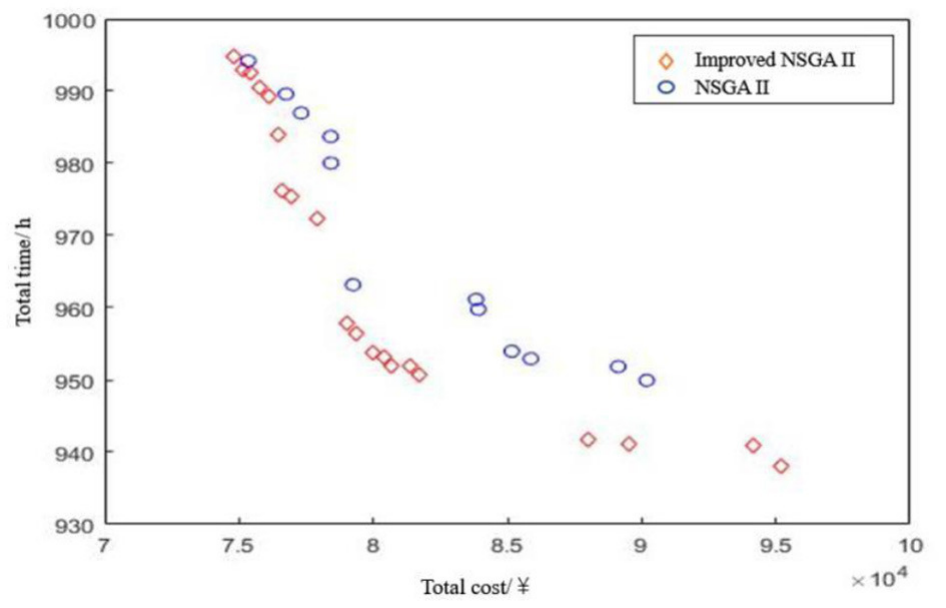
Two multi objective genetic algorithms.

In the context of public health emergencies in relation 3D printing equipment resources, the matching strategy is an NP-hard problem, and the true Pareto front is unknown. In order to verify that the validity of the NSGA-II algorithm had improved, using the convergence and running time of the algorithm as the evaluation standards, the results of both the new and traditional NSGA-II algorithm under the same parameters and over 10 runs each were analyzed. Using the above experimental data to compare the NSGA-II algorithms crossover probability was set at 0.9, mutation probability at 0.05, the population size at 100, and the largest iterative algebra at 500. The simulation environment was the Intel (R) Core (TM) i5-4460S, CPU@2.50GHz, RAM4GB, system, Windows 10 and Simulation platform Matlab2015a. Table 3 shows the results of the comparison between the improved NSGA-II algorithm and the traditional NSGA-II algorithm, which were run 10 times each. Using the traditional NSGA-II algorithm and the improved the NSGA-II algorithm, the optimal Pareto solutions were obtained, as shown in Figure 5.

(1) In terms of the convergence of the algorithm, it can be seen from Table 5 that the average cost calculated by the improved NSGA-II algorithm is lower than that of the traditional NSGA-II algorithm, that is, the improved NSGA-II algorithm can obtain a solution with higher user satisfaction at the same cost. It can be intuitively seen from Figure 5 that Pareto solution obtained by the improved NSGA-II algorithm under the same conditions is better than that obtained by the traditional NSGA-II algorithm. In addition, the optimal cost obtained by the improved NSGA-II algorithm is also slightly higher than that of the traditional NSGA-II algorithm, indicating that its search space is larger. In summary, the Pareto frontier obtained by the improved NSGA-II algorithm is better, and its convergence is better than that of the traditional NSGA-II algorithm.

**Table 5:** Improved NSGA-II algorithm performance comparison.

**The optimization goal**	**Cost of emergency supplies × 10^4^/Yuan**		**Emergency supply time /h**		**Algorithm running time/s**	
	**Average**	**Optimal**	**Average**	**Optimal**	**Average**	**Optimal**
**Improved NSGA-II algorithm**	8.314	7.482	966	938	11.28	10.85
**NSGA-II algorithm**	8.500	7.560	972	950	12.03	11.82

(2) In regard to the algorithm running time, Table 5 shows that the traditional NSGA-II algorithm had an average running time of 12.03 s, while the improved NSGA-II algorithm had an average running time of 11.28 s, thus providing a time reduction of 6.2%. In addition, the optimal operation time of the improved NSGA-II algorithm was 10.85 s, while that of the traditional NSGA-II algorithm was 11.82 s, thus improving the speed of the NSGA-II algorithm.

In conclusion, compared with the traditional NSGA-II algorithm, the improved NSGA-II algorithm has a slight improvement in overall performance. Usage of the optimal solution set in the improved NSGA-II algorithm is shown in Table 6.

**Table 6:** Improved NSGA-II algorithm and Pareto optimal solution (partial).

**Pareto optimal solution**	**Total cost of emergency supplies/Yuan**	**Total time for emergency supplies /h**
[13,5,13,13,4,15,12,11,18,13,16,9,13,20,4,1,3,12,4,13,19,17,1,16,8,12,18,11,16,19,8,13,12,3,12,9,18,11,13,3,13,18,19, 13,11,5,9, 3,1,4]	74,815	995
[13,5, 13,13,4,15,12,11,18,13,16,9,13,19,4,1,3,12,4,13,19, 17,1,16,8,12,18,11,16,19,8,13,12,3,12,9,18,11,13,3,13,18, 19,13,11,5,9,3,1,4]	75,153	992
[13,5,13,13,4,16,12,11,18,13,16,9,13,20,4,1,3,12,4,13,19,17,1,16,8,12,18,11,16,19,8,13,12,3,12,9,18,11,13,3,13,18,19,13,11,5,9,3,1,4]	75,762	990
[13,5,13,13,4,15,12,11,18,13,16,8,13,19,4,1,3,12,4,13,20,17,1,16,10,12,18,11,16,19,8,13,12,3,12,9,18,11,13,4,13,18,19,13,11,6, 9,3,1,4]	76,458	983
[13,5,13,13,4,15,12,11,18,13,16,8,13,19,4,2,3,13,4,13,20,16,1,16,10,12,18,11,16,19,8,13,12,3,11,9,18,11,13,4,13,18,18,13,11,6, 9,3,1,4]	76,571	976
[13,5,13,13,4,15,12,11,18,13,16,8,13,19,4,2,3,13,4,13,20, 16,1,17,10,12,18,11,16,19,8,13,12,3,11,9,18,11,13,4,13,18,18,13,11,6,9,2,1, 4]	77,945	972
[13,5,13,13,4,17,12,11,18,13,16,8,13,19,4, 1,3,13,4,13,19,17,1,17,7,11,18,11,16,20,8,13,13,3,13,9,18,11,13,2,13,20,19,13,11,6,9,4,1,4]	79,042	957
[13,5,13,13,4,17,12,11,17, 13,16,8,13,19,4,1,3,13,4,13,19,17,1,17,6,11,18,11,16,20,8,13,13,3,13,9,18,11,13,2,13,20,19,13,11,6,9,4,1,4]	80,704	952
[13,5,13,13,4,17,12,11,17,13,16,8,13,19,4,1,3,13,4,13,19,17,1,17,6,11,18,11,16,20,8,13,13,3,13,9,18,11,13,2,13,20,19,13,10,6, 9,4,1,4]	81,682	950
[7,6,17,5,3,4,10,6,10,6,16,6,13,16,13,17,2,17,13,10,12,8,1,4,19,8,4,17,13,13,16,17,17,2,6,13,13, 3,11,17,2,10,6,19,6,17,11,16,11,14]	94,178	941
[8,6,17,6,3,3,10,6,10, 6,16,6,13, 16,13,17,2,17, 13,10,13, 7,1,4,19,8,4,17,13,13,16,17,17,2,6,13,13,3,11,17,2,10,7,19,6,17,11,17,11,14]	95,199	938

## Conclusion

In this study, combining with current public health emergencies, this paper proposes the scheduling and distribution scheme of emergency supplies produced by 3D printing technology. In terms of the model, a 3D printing production emergency material scheduling model was built to solve the resource matching problem between supply and demand and maximize the production cost and time benefit. In algorithm, orthogonal crossover strategy was applied to algorithm in the process of the cross, and introducing the adaptive hybrid mutation operator, get an improved the NSGA-II algorithm, and compared with the traditional NSGA-II algorithm, the results show that the improved algorithm has higher precision and faster convergence speed, convergence of optimization result is closer to the global optimal solution, and more effectively solve the multi-objective problem. It is proved that the improved NSGA-II algorithm can obtain better performance, which has a certain theoretical significance. We suggest that, under the background of Covid-19 epidemic, the study on supply and demand matching of 3D printing emergency supplies can solve the problem of concealment of some organizations in material distribution. Meanwhile, managers can reasonably allocate emergency supplies according to different disaster situations in each disaster area, which is of certain practical significance.

This study solves the scheduling problem of 3D-printed emergency supplies in the event of public health emergencies, but the workflow of emergency supplies requirements selected by the model is static. However, the most significant impact of 3D printing on users' emergency supplies is the dynamic change in demand; Therefore, the next step will be to conduct research on 3D printing scheduling of dynamic emergency supplies for public health emergencies under the same constraint conditions as the optimization objective.

## Data Availability Statement

The original contributions presented in the study are included in the article/supplementary material, further inquiries can be directed to the corresponding author/s.

## Author Contributions

JH came up with the topic idea selection and structured the manuscript ideas. GL designed the research plan, constructed the model, and wrote the thesis. TM analyzed the selected articles to verify their relevance for the study. TL wrote conclusion and collect data. All authors contributed to the article and approved the submitted version.

## Conflict of Interest

The authors declare that the research was conducted in the absence of any commercial or financial relationships that could be construed as a potential conflict of interest.

## References

[1] Oladapo BI Ismail SO Afolalu TD Olawade DB Zahedi M. Review on 3D printing: fight against COVID-19. Mater Chem Phys. (2021) 258:95–110. 10.1016/j.matchemphys.2020.123943 33106717 PMC7578746

[2] Wang Q Zhang T Zhu H Wang Y Liu X Bai G. Characteristic of and public health emergency responses to COVID-19 and H1N1 out-breaks: a case-comparison study. Int J Environ Res Public Health. (2020) 7:12–23. 10.3390/ijerph17124409 PMC7344548 32575492

[3] Corsini L Aranda-Jan CB Moultrie J. The impact of 3D printing on the humanitarian supply chain. Prod Plan Control. (2020) 78:15–30. 10.1080/09537287.2020.1834130

[4] Campbell I Bourell D Gibson I. Additive manufacturing: rapid prototyping comes of age. Rapid Prototyping J. (2012) 18:255–8. 10.1108/13552541211231563

[5] Ngo TD Kashani A Imbalzano G Nguyen KTQ Hui D. Additive manufacturing (3D printing): a review of materials, methods, applications and challenges. Compos Pt B Eng. (2018) 143:172–96. 10.1016/j.compositesb.2018.02.012

[6] Liu W Wang Q Mao Q Wang S Zhu D. A scheduling model of logistics service supply chain based on the mass customization service and uncertainty of FLSP's operation time. Transport Res Pt E Transp Res E Log. (2015) 83:189–215. 10.1016/j.tre.2015.09.003

[7] Mourtzis D Doukas M Psarommatis F. A toolbox for the design, planning and operation of manufacturing networks in a mass customisation environment. J Manuf Syst. (2015) 36:274–86. 10.1016/j.jmsy.2014.06.004

[8] Li Q Kucukkoc I Zhang DZ. Production planning in additive manufacturing and 3D printing. Comput Oper Res. (2017) 83:157–72. 10.1016/j.cor.2017.01.013

[9] Khajavi SH Partanen J Holmström J. Additive manufacturing in the spare parts supply chain. Comput Ind. (2014) 65:50–63. 10.1016/j.compind.2013.07.008

[10] Guoqing W Yingzhou H Weinan Z Chenguang S Hongsong H. Research status and development trend of laser additive manufacturing technology. In: 2017 4th International Conference on Information Science and Control Engineering. Changsha (2017). p. 1210–13.

[11] Sheu JB. Post-disaster relief–service centralized logistics distribution with survivor resilience maximization. Transport Res B Meth. (2014) 68:288–314. 10.1016/j.trb.2014.06.016

[12] Zhan SL Liu N Ye Y. Coordinating efficiency and equity in disaster relief logistics via information updates. Int J Syst Sci. (2014) 45:1607–21. 10.1080/00207721.2013.777490

[13] Lu CC Ying KC Chen H-J. Real-time relief distribution in the aftermath of disasters - a rolling horizon approach. Transport Res Pt E Transp Res E Log. (2016) 93:1–20. 10.1016/j.tre.2016.05.002

[14] Zhou Y Liu J Zhang Y Gan X. A multi-objective evolutionary algorithm for multi-period dynamic emergency resource scheduling problems. Transport Res Pt E Logs Transport Rev. (2017) 99:77–95. 10.1016/j.tre.2016.12.011

[15] Alem D Clark A Moreno A. Stochastic network models for logistics planning in disaster relief. Eur J Oper Res. (2016) 255:187–206. 10.1016/j.ejor.2016.04.041

[16] Ekici A Keskinocak P Swann JL. Modeling influenza pandemic and planning food distribution. Mandsom Manuf Serv Op. (2014) 16:11–27. 10.1287/msom.2013.0460

[17] He Y Liu N. Methodology of emergency medical logistics for public health emergencies. Transport Res Part E Transport Res E Log. (2015) 79:178–200. 10.1016/j.tre.2015.04.007 32288598 PMC7147567

[18] Liu M Zhang D. A dynamic logistics model for medical resource allocation in epidemic control with demand forecast updating. J Oper Res Soc. (2016) 67:841–5. 10.1057/jors.2015.105

[19] Whybark DC. Issues in managing disaster relief inventories. Int J Prod Econ. (2007) 108:228–35. 10.1016/j.ijpe.2006.12.012

[20] Chakravarty AK. Humanitarian relief chain: rapid response under uncertainty. Int J Prod Econ. (2014) 151:146–57. 10.1016/j.ijpe.2013.10.007

[21] Sheu JB Pan C. Relief supply collaboration for emergency logistics responses to large-scale disasters. Transport A Transport Sci. (2015) 11:210–42. 10.1080/23249935.2014.951886

[22] Wang X Li F Liang L Huang Z Ashley A. Pre-purchasing with option contract and coordination in a relief supply chain. Int J Prod Econ. (2015) 167:170–6. 10.1016/j.ijpe.2015.05.031

[23] Luo X Zhang L Ren L Lali Y. A dynamic and static data based matching method for cloud 3D printing. Robot Comput Integr Manuf . (2020) 61:101858. 10.1016/j.rcim.2019.101858

[24] Deb K Pratap A Agarwal S Meyarivan T. A fast and elitist multi-objective genetic algorithm: NSGA-II. IEEE T Evolut Comput. (2002) 6:182–97. 10.1109/4235.996017

